# Study of Viscoelastic Characteristics of Polyacrylamide Solutions in Polymer Flooding of Heterogeneous Reservoirs

**DOI:** 10.3390/gels12050367

**Published:** 2026-04-28

**Authors:** Inzir Ramilevich Raupov, Ahmed Kone, Alexey Feinberg

**Affiliations:** Department of Oil and Gas Fields Development and Operation, Empress Catherine II Saint Petersburg Mining University, 2, 21st Line, 199106 St. Petersburg, Russia; raupov_@pers.spmi.ru (I.R.R.); morokov_aa@pers.spmi.ru (A.F.)

**Keywords:** rheology, enhanced oil recovery (EOR), polymer injection, relaxation time, core flooding

## Abstract

This study addresses the need for enhanced oil recovery (EOR) in mature reservoirs, particularly in Russian oil fields that have undergone prolonged production and exhibit declining performance. Among EOR techniques, polymer flooding remains one of the most widely applied and effective methods following conventional waterflooding. In this work, the rheological and viscoelastic behavior of partially hydrolyzed polyacrylamide (HPAM) solutions and their impact on oil displacement efficiency in heterogeneous reservoirs were investigated. Two polymers with different molecular weights were evaluated using steady shear, oscillatory rheology, and one-dimensional core flooding experiments. The results revealed pronounced shear-thinning behavior, with viscosity increasing with polymer concentration and molecular weight. Viscoelasticity was observed only for the high-molecular-weight polymer, characterized by a well-defined linear viscoelastic region and relaxation behavior sensitive to pore size, salinity, and temperature. Core flooding experiments showed that waterflooding recovered 30–31% OOIP, while high-molecular-weight polymer injection increased recovery to ~62% OOIP. In contrast, low-molecular-weight polymer yielded only ~40% OOIP, whereas a combined injection strategy achieved up to 74–76% OOIP. These findings highlight the critical role of polymer molecular weight and viscoelasticity in improving sweep efficiency and enhancing oil recovery in heterogeneous reservoirs.

## 1. Introduction

Fossil fuels remain the dominant source of global energy supply despite the rapid development of renewable energy technologies in recent decades [1,2,3]. The continuous growth of the global population, together with increasing industrial activity and transportation demand, has led to a steady rise in energy consumption worldwide. Although renewable sources such as solar and wind energy are expanding, their current contribution is still insufficient to fully replace conventional hydrocarbons. Consequently, crude oil continues to play a critical role in ensuring global energy security and supporting economic development.

However, many large conventional oil fields are currently experiencing progressive depletion. The deterioration of reserve structures in several producing regions, including Russia and other major oil-producing countries, has significantly affected production stability [4,5,6,7]. In mature reservoirs, primary and secondary recovery methods typically extract only 30–40% of the original oil in place, leaving a substantial fraction of hydrocarbons trapped within the porous medium. Therefore, the development and implementation of advanced recovery techniques have become essential to maintain production levels and improve the overall recovery factor.

In this context, tertiary recovery methods, commonly referred to as Enhanced Oil Recovery (EOR), have gained considerable attention over the past decades. Significant progress has been made in the development of EOR technologies, particularly in China, where large-scale projects have been implemented to enhance production from mature oil fields [8,9,10]. These techniques aim to mobilize the remaining oil trapped after conventional water flooding by altering fluid properties, improving sweep efficiency, or modifying rock–fluid interactions. Among the different EOR methods, chemical approaches have proven particularly effective for improving displacement efficiency in heterogeneous reservoirs.

Polymer flooding is one of the most widely applied chemical EOR techniques and has been successfully implemented in numerous oil fields worldwide for several decades [11,12,13]. Field applications have demonstrated that polymer flooding can significantly increase oil recovery compared with conventional water flooding [14]. By modifying the rheological properties of the injected fluid, polymer flooding improves sweep efficiency and reduces the unfavorable mobility ratio between injected water and reservoir oil.

The principle of polymer flooding consists of increasing the viscosity of the injected water in order to reduce the mobility ratio and stabilize the displacement front within the reservoir [14,15,16,17]. A more favorable mobility ratio leads to a more uniform displacement of oil and limits the formation of viscous fingering, which often occurs during conventional water flooding in heterogeneous formations. As a result, polymer flooding enhances areal and vertical sweep efficiency, allowing a larger portion of the reservoir to be effectively drained.

Among the different polymers used in EOR processes, partially hydrolyzed polyacrylamide (HPAM) is currently the most widely applied synthetic polymer [18,19,20]. HPAM is a copolymer obtained through the partial hydrolysis of polyacrylamide, which provides suitable rheological properties for mobility control during injection. Despite their widespread use and relatively good performance, HPAM-based polymer solutions remain sensitive to reservoir conditions such as high temperature, high salinity, water hardness, and strong shear stresses encountered during injection and flow through porous media [21,22,23]. These factors may lead to polymer degradation, loss of viscosity, or structural modifications of the polymer chains, thereby reducing the efficiency of the flooding process.

Despite significant progress in the understanding of polymer flooding mechanisms, several aspects related to polymer flow in porous media remain insufficiently understood. In particular, polymer injectivity, in-situ viscosity behavior, and the potential reduction of residual oil saturation (Sor) associated with viscoelastic effects have attracted increasing research interest in recent years [24,25,26]. Studying viscoelastic behavior is particularly important for gel systems in which polymer crosslinking with polyvalent metal ions induces a transition from viscoplastic to viscoelastic behavior. Such investigations enable the identification of the onset and completion of the gelation process and facilitate the optimization of the required amount of gelling agent in polymer formulations. Furthermore, the use of viscoelastic polymers in oil displacement processes with polymer solutions can generate a high resistance factor and redirect filtration flows toward poorly drained oil-saturated zones, similarly to the mechanisms observed in conformance control. Experimental studies have suggested that the elastic properties of polymer solutions may contribute to the mobilization of trapped oil droplets by generating additional microscopic forces during flow through pore throats. However, the mechanisms governing these effects and their dependence on reservoir and fluid parameters remain subjects of ongoing investigation.

The objective of this study is therefore to investigate the relationship between polymer viscoelasticity—characterized by the relaxation time—and several key parameters, including polymer concentration, pore diameter, sodium chloride concentration, and temperature. Understanding the influence of these parameters on polymer viscoelastic behavior is essential for optimizing polymer selection and injection strategies in EOR operations. The results of this work aim to provide further insight into the mechanisms controlling polymer flow in porous media and to contribute to improving the efficiency of polymer flooding processes in complex reservoir conditions.

## 2. Results and Discussion

### 2.1. Shear Characterization: Effect of Shear Rate and Pore Diameter on Viscosity

The resulting flow curves (viscosity vs. shear rate) were analyzed to characterize the non-Newtonian behavior of the polymer solutions. The investigated shear rate range (1–100 s^−1^) is particularly relevant for EOR applications, where the lower limit represents reservoir flow conditions, and the upper limit corresponds to high-shear regions near the wellbore and during injection [27].

It should be noted that results for the low-molecular-weight polymer (HPAM 3230S) are not presented, as the corresponding viscosity curves exhibited trends similar to those of the high-molecular-weight polymer (HPAM 3630S), but with consistently lower viscosity values across the entire shear rate range. Therefore, only the high-molecular-weight polymer results are shown for clarity.

**Figure 1:**
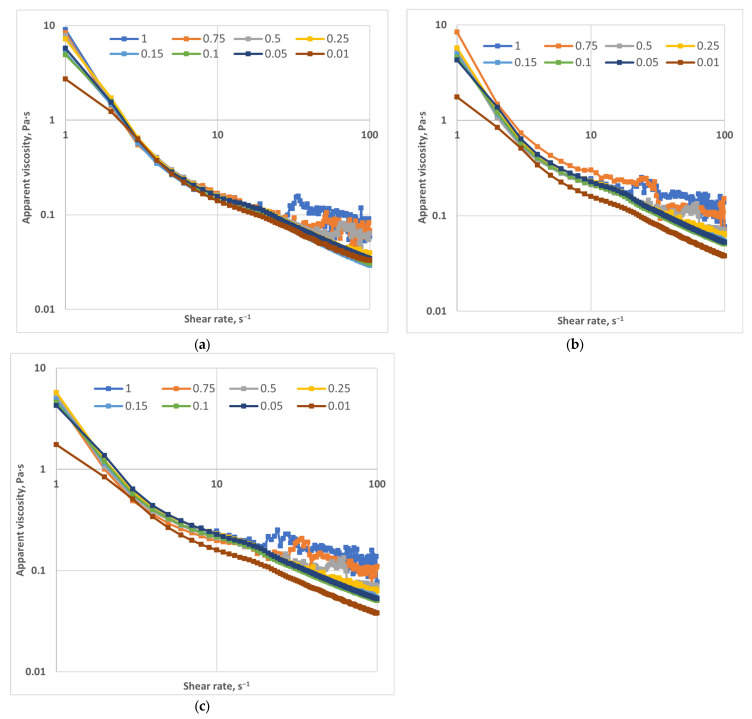
Effect of shear rate and pore diameter on viscosity: (**a**) 5000 ppm; (**b**) 7500 ppm; (**c**) 10,000 ppm. (T = 22 °C).

A steady shear test was performed over the range of 1–100 s^−1^. Figure 1 illustrates the variation of viscosity with shear rate for polymer concentrations of 5000, 7500, and 10,000 ppm, measured at different plate gaps (0.01 to 1 mm) under a salinity of 3 wt% NaCl. All samples exhibited typical shear-thinning behavior, characterized by a decrease in viscosity with increasing shear rate due to progressive alignment and deformation of polymer chains [28,29,30].

The high-molecular-weight polymer showed higher viscosity values, particularly at low shear rates, which is representative of flow conditions in porous reservoir media. This behavior is attributed to its higher molecular weight and enhanced intermolecular interactions [31,32,33]. Increasing polymer concentration led to a significant rise in viscosity, with a more pronounced effect observed at 7500 and 10,000 ppm.

In addition, increasing the plate gap resulted in higher apparent viscosity values. At high shear rates and large gaps, a viscosity plateau was observed, indicating a transition toward Newtonian behavior associated with the progressive breakdown of the polymer structure.

### 2.2. Oscillatory Rheology

#### 2.2.1. Amplitude Sweep

It is important to note that the viscoelastic analysis presented herein applies exclusively to the high-molecular-weight polymer (HPAM 3630S). Under the investigated concentration range, the lower-molecular-weight polymer (HPAM 3230S, Mw = 6–8 × 10^6^ g/mol) did not exhibit measurable viscoelastic behavior, with G″ consistently dominating over G′ across all tested conditions. This indicates a predominantly viscous response and the absence of an entangled polymer network capable of storing elastic energy. Such behavior is consistent with previous studies demonstrating that viscoelasticity in HPAM solutions strongly depends on molecular weight and concentration, with lower-molecular-weight polymers failing to develop significant elastic properties at moderate concentrations [34,35,36].

**Figure 2:**
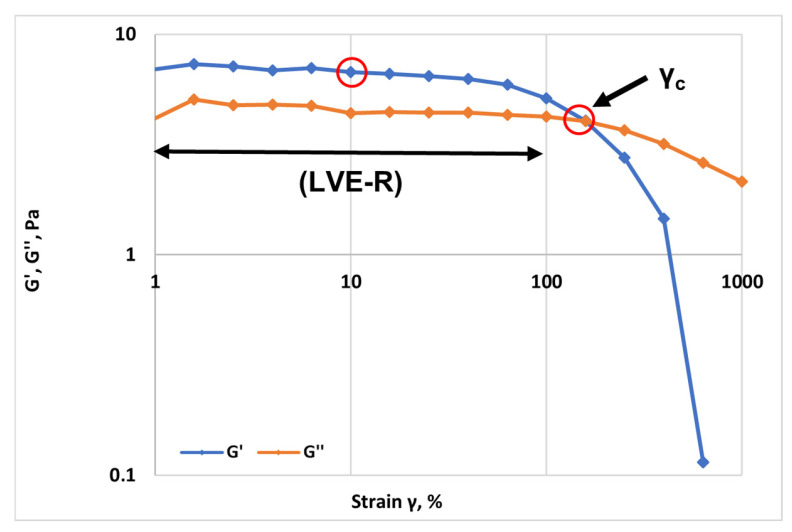
Amplitude sweep test of the polymer solution at 5000 ppm at 25 °C (gap = 1 mm): storage modulus (G′) and loss modulus (G″) as a function of shear strain (γ).

The amplitude sweep results (Figure 2) demonstrate a well-defined linear viscoelastic region (LVER) in which both the storage modulus (G′) and the loss modulus (G″) remain essentially independent of strain amplitude up to approximately 100% deformation. Within this regime, G′ consistently exceeds G″, indicating a predominantly elastic, solid-like response characteristic of an entangled polymer network with sufficient structural integrity to store mechanical energy. For the characterized polymer solutions, a strain amplitude of γ = 10% was determined to lie safely within the LVE-R and was subsequently adopted for frequency sweep analysis. Beyond the critical strain (γ_c_), both moduli progressively decrease, marking the onset of nonlinear viscoelastic behavior associated with structural rearrangement, chain alignment, and progressive disentanglement under increasing deformation. The sharper decline of G′ compared to G″ suggests that the elastic network is more sensitive to strain-induced disruption than viscous dissipation mechanisms. At high strain amplitudes, the pronounced reduction in G′ and the eventual predominance of viscous contributions reflect substantial breakdown of the transient polymer network.

This strain-softening behavior is typical of concentrated viscoelastic polymer solutions and confirms that the mechanical stability of the system is governed by reversible intermolecular entanglements that are progressively disrupted under large deformations.

#### 2.2.2. Frequency Sweep

**Figure 3:**
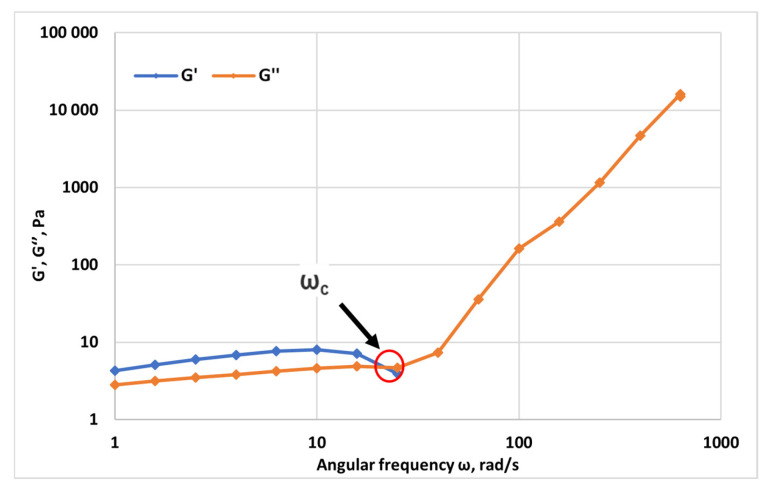
Frequency-dependent viscoelastic response of polymer solution at 5000 ppm at 25 °C (gap = 1 mm) showing storage modulus (G′), loss modulus (G″), and crossover frequency (ω_c_).

The frequency sweep results (Figure 3) illustrate the viscoelastic behavior of the HPAM 3630S solution (5000 ppm) over the investigated angular frequency range (1–1000 rad·s^−1^). At low frequencies, the storage modulus (G′) slightly exceeds the loss modulus (G″), indicating a weak elastic-dominated response associated with long relaxation times and an entangled polymer network capable of sustaining stress over extended timescales. As the angular frequency increases, both moduli evolve differently, and a crossover point (ω_c_) is observed where G′ = G″. This crossover frequency marks the transition from elastic-dominated behavior at long timescales (low ω) to viscous-dominated behavior at shorter timescales (high ω), reflecting the characteristic relaxation dynamics of the system. Beyond ω_c_, G″ increases more rapidly than G′, and at high frequencies, the viscous contribution becomes predominant, indicating that molecular motions are increasingly governed by local chain dynamics rather than network elasticity. The position of ω_c_ provides a direct estimate of the principal relaxation time (λ = 1/ω_c_), which characterizes the longest relaxation mode of the entangled polymer structure. Overall, the observed frequency-dependent moduli confirm the typical behavior of concentrated viscoelastic polymer solutions, where the interplay between chain entanglement, segmental mobility, and hydrodynamic interactions governs the transition from solid-like to liquid-like response.

### 2.3. Effect of Polymer Concentration and Pore Diameter on Relaxation Time

**Figure 4:**
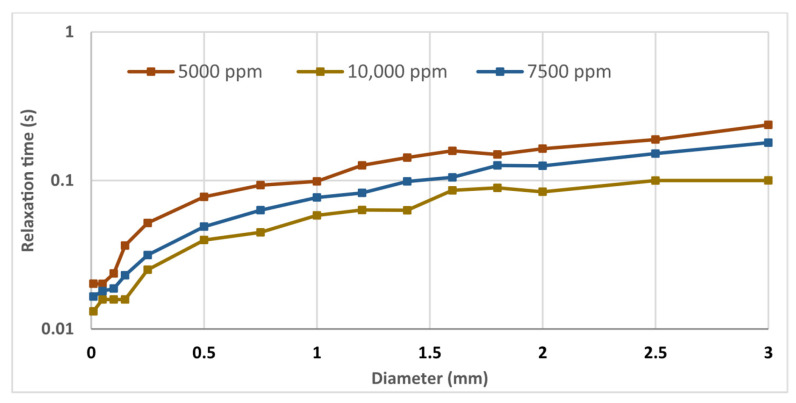
Effect of polymer concentration and pore diameter on relaxation time (T = 25 °C).

Figure 4 illustrates the relationship between polymer concentration, pore diameter, and relaxation time, highlighting the combined effect of these two parameters on the viscoelastic dynamics of the polymer system. Indeed, the progressive increase in relaxation time with increasing gap indicates a confinement-to-bulk transition in the viscoelastic response. Under confined conditions, reduced entanglement density and wall-induced chain orientation promote faster stress relaxation, in agreement with previous studies on confined polymer dynamics [37,38]. Interestingly, the highest concentration (10,000 ppm) displays shorter relaxation times than lower concentrations, deviating from classical entanglement scaling expectations. In partially hydrolyzed polyacrylamide systems, enhanced ionic screening at elevated polymer loadings can induce coil contraction, thereby reducing effective chain overlap and altering the relaxation spectrum [39]. Additionally, shear-induced alignment and transient disentanglement under rheometric conditions may preferentially activate faster relaxation modes [40]. The possible formation of transient associative domains or structural heterogeneities at high concentration may further modify stress dissipation pathways [41]. These combined effects suggest that the dominant relaxation process shifts from entanglement-controlled reptation to faster local or screened modes at the highest concentration investigated.

### 2.4. Effect of Salt Concentration on Relaxation Time

**Figure 5:**
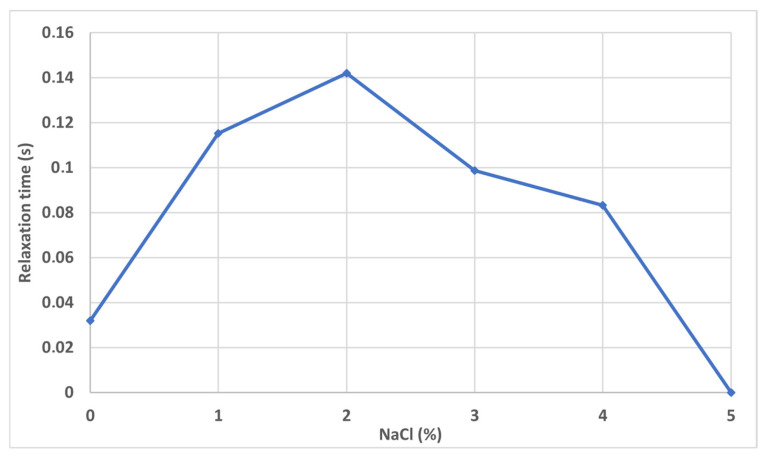
Influence of salt concentration on the relaxation time of high molecular weight polymer solutions. (T = 25 °C).

Figure 5 shows the effect of salt concentration on a 5000 ppm HPAM 3630S solution. The gap between the rheometer planes is 1 mm at 25 °C.

The non-monotonic evolution of the relaxation time of the polymer solution with increasing NaCl concentration is consistent with the well-established polyelectrolyte behavior of partially hydrolyzed polyacrylamide. At very low salinity, electrostatic repulsion between ionized carboxylate groups along the backbone promotes chain expansion, but incomplete charge screening and intermolecular association can limit effective elastic response. Upon addition of moderate salt concentrations (≈1–2 wt% NaCl), electrostatic screening reduces long-range intramolecular repulsion while maintaining sufficient chain extension and entanglement density, leading to enhanced coil elasticity and a maximum in relaxation time. This behavior aligns with classical polyelectrolyte theory and experimental observations showing that moderate ionic strength can increase viscoelastic relaxation by optimizing hydrodynamic volume and transient network formation [42,43]. Similar salinity-dependent maxima in viscoelastic parameters for HPAM and related polyacrylamides have been reported in rheological studies relevant to enhanced oil recovery, where intermediate salt levels enhance elastic effects before coil contraction dominates [44,45]. At higher NaCl concentrations (>3–4 wt%), stronger charge screening and ion–polymer interactions induce significant coil contraction, reduced overlap concentration effectiveness, and partial disruption of entanglement networks, thereby decreasing relaxation time and ultimately suppressing measurable elasticity at very high salinity. This salinity-induced coil shrinkage and reduction in viscoelastic relaxation has been widely documented for anionic polyacrylamides and other flexible polyelectrolytes [46,47]. Overall, the observed peak in relaxation time at intermediate NaCl concentration reflects the balance between electrostatic expansion and ionic screening effects governing chain conformation, entanglement density, and transient network dynamics in HPAM solutions.

### 2.5. Effect of Temperature on Relaxation Time

**Figure 6:**
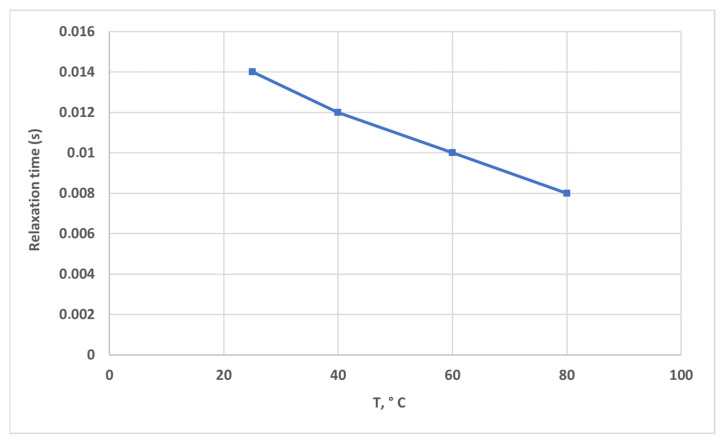
Temperature influence on relaxation time of high molecular weight polymer solutions.

In this test, a solution of HPAM 3630S with a concentration of 5000 ppm was subjected to a temperature ramp with a gap of 1 mm between the parallel plates. The results show that the relaxation dynamics of polymer solutions are strongly influenced by temperature, a key observation in polymer rheology. In our experiment, the relaxation time of the polymer solution systematically decreases with increasing temperature, consistent with the enhanced molecular mobility and accelerated chain dynamics at higher thermal energy (Figure 6). This temperature sensitivity has been previously documented in aqueous polymer systems such as hydrolyzed polyacrylamide, where relaxation times were quantified over a temperature range and modeled using time–temperature shift approaches like the Williams–Landel–Ferry equation. Likewise, in supramolecular polymer solutions, the terminal relaxation time exhibits an exponential dependence on temperature, underscoring the thermally activated nature of the molecular relaxation processes [48]. Rheological studies on concentrated polymer solutions also show that shear and stress relaxation functions shift systematically with temperature according to time–temperature superposition principles, leading to shorter characteristic relaxation times at elevated temperatures [49]. Beyond oscillatory shear, extensional rheometry of viscoelastic polymer solutions reveals measurable relaxation times that vary with thermal conditions, reinforcing that increased thermal motion facilitates faster relaxation dynamics [50]. Moreover, time–temperature scaling analyses for linear viscoelastic polymer solutions have illustrated that temperature acts to compress relaxation spectra toward shorter times, in line with classical viscoelastic models and experiments [51]. Together, these observations corroborate our finding of decreasing relaxation time with increasing temperature and situate it within the broader framework of polymer viscoelasticity [52].

### 2.6. Results of Core Samples Flooding Experiments

Polymer flooding is widely recognized as one of the most effective and economically viable enhanced oil recovery (EOR) methods following conventional waterflooding. To evaluate the incremental oil recovery associated with polymer injection, a comparative experimental study of waterflooding and polymer flooding was conducted under controlled laboratory conditions. The waterflooding experiment was designed to replicate the same methodological framework as the polymer flooding tests in order to minimize experimental uncertainties and ensure comparability of results. Specifically, the waterflooding process consisted of an initial injection of 0.8 pore volumes (PV), followed by an additional 1 PV corresponding to the polymer slug volume used in polymer flooding experiments, and concluded with a 0.5 PV water post-flush, resulting in a total injected volume of approximately 2.3 PV. The experiments were performed at a temperature of 57 °C and a constant flow rate of 1 mL/min, using core samples initially saturated with oil of viscosity 75 mPa·s.

#### 2.6.1. Establishment of Baseline Conditions Using Waterflooding

**Figure 7:**
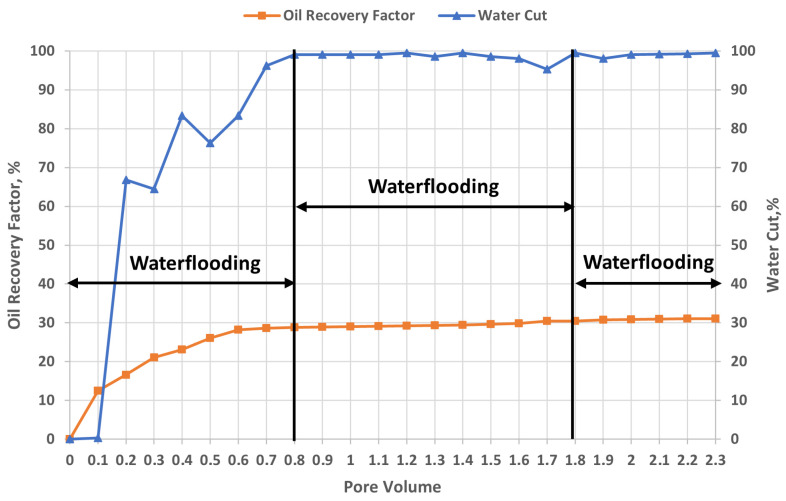
Oil recovery factor and water cut response during waterflooding (T = 57 °C).

Figure 7 presents the evolution of oil recovery and water cut as a function of injected water volume during waterflooding. The results of the one-dimensional core displacement experiments demonstrate the characteristic features of immiscible oil displacement in porous media and are consistent with classical flow theory. The experiments were conducted using two core samples with contrasting permeability (low- and high-permeability media), allowing assessment of the impact of heterogeneity on displacement efficiency. Oil production begins simultaneously in both cores upon water injection; however, the contribution from the high-permeability core dominates, while production from the low-permeability core is limited due to higher hydraulic resistance. As injection proceeds, the low-permeability zone becomes progressively involved in the displacement process, and from approximately 0.9 PV, oil production from both cores tends to equilibrate, indicating partial flow redistribution and mobilization of previously unswept regions.

At the early stage of injection (up to approximately 0.2 PV), a near-piston-like displacement regime is observed, characterized by negligible water cut and a rapid increase in oil recovery to 15–20%, reflecting high microscopic displacement efficiency and a relatively stable displacement front. This behavior is consistent with the classical Buckley–Leverett theory [53]. However, in the range of approximately 0.25–0.35 PV, a sharp increase in water cut is observed—from near zero to 60–80%—indicating early water breakthrough and the transition to two-phase flow. This premature breakthrough is attributed to an unfavorable mobility ratio (M > 1), further amplified by permeability contrast between the cores. As a result, preferential flow paths develop in the high-permeability zones, leading to channeling and reduced sweep efficiency. This behavior is characteristic of hydrodynamic instability and viscous fingering, as widely reported in the literature [54,55].

In the post-breakthrough region (0.4–0.8 PV), water cut rapidly approaches unity, while the increase in oil recovery slows significantly, reaching approximately 28–29%. This reflects the formation of dominant flow channels in high-permeability regions and poor macroscopic sweep efficiency, with significant bypassing of oil in low-permeability zones. At later stages of injection (beyond ~0.8 PV up to 2.3 PV), the system reaches a quasi-steady state, characterized by water cut levels of 98–100% and an oil recovery plateau of approximately 30–31%. This plateau corresponds to the attainment of residual oil saturation (S_or_), beyond which further water injection does not significantly increase oil recovery due to capillary trapping and limited microscopic displacement efficiency.

Overall, the relatively low ultimate oil recovery observed in these experiments is typical of conventional waterflooding under conditions of unfavorable mobility control and pronounced reservoir heterogeneity. The combined effects of permeability contrast, viscous instability, and capillary forces significantly limit both macroscopic and microscopic displacement efficiency. These results highlight the limitations of secondary recovery methods and justify the application of enhanced oil recovery techniques, such as polymer flooding (to improve mobility control) or surfactant injection (to reduce interfacial tension), in order to enhance oil recovery in heterogeneous reservoir systems.

#### 2.6.2. Effect of High-Molecular-Weight Polymer on Heterogeneous Oil Reservoirs

**Figure 8:**
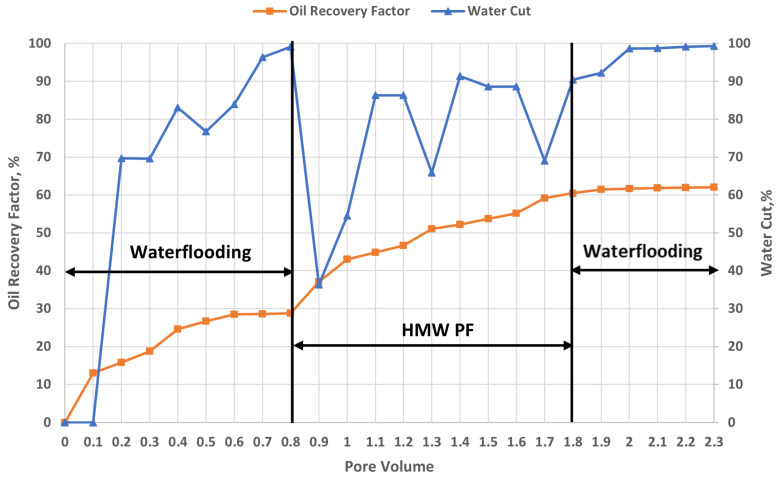
Oil recovery factor and water cut response during Flopaam 3630S flooding (T = 57 °C).

Figure 8 presents the evolution of the oil recovery factor and water cut as a function of injected pore volumes (PV), allowing the evaluation of the impact of high molecular weight polymer injection in a heterogeneous reservoir. It should be emphasized that the initial waterflooding stage (0–0.8 PV) was conducted under conditions strictly identical to the baseline experiments, using the same crude oil, temperature, and synthetic brine. Therefore, the results obtained in this interval correspond to conventional waterflooding behavior and can be considered as a reference for subsequent analysis.

It is also observed that, during waterflooding, oil production begins immediately upon synthetic brine injection in the high-permeability core, whereas production from the low-permeability core starts later, around 0.5 PV. From this point onward, oil production from the low-permeability core progressively increases as pressure gradients build up and partial crossflow occurs. This delayed response confirms the poor initial sweep of low-permeability regions under waterflooding conditions.

Upon transition to polymer injection (starting at 0.8 PV), a marked change in displacement behavior is observed. Water cut drops sharply (from approximately 100% to ~36%), while oil recovery increases significantly. Between 0.8 and 1.8 PV, the recovery factor rises from ~29% to ~61%, corresponding to an incremental oil recovery of approximately 32% of OOIP during polymer flooding. This stage represents the dominant contribution to total recovery improvement. Notably, oil production from the low-permeability core reaches a peak shortly after the start of polymer injection, around 0.9 PV. At this point, oil production from the low-permeability core temporarily exceeds that of the high-permeability core, coinciding with a reduction in water cut (from ~95–100% down to ~36%). This peak reflects a transient but significant improvement in sweep efficiency due to flow redistribution.

This behavior can be explained by the combined effects of mobility control and viscoelasticity of the high molecular weight polymer. The increased viscosity of the injected fluid reduces the mobility ratio, limiting channeling in high-permeability zones and forcing the displacing fluid into previously unswept, lower-permeability regions. In addition, the large molecular size and coil dimensions of the polymer molecules, which are comparable to pore throat sizes in the porous medium, contribute to a partial blocking effect in high-permeability channels. This enhances flow diversion toward tighter zones. Furthermore, the viscoelastic properties of the polymer play a key role at the microscopic scale: elastic stresses generated under extensional flow conditions promote the deformation and mobilization of trapped oil ganglia, leading to additional oil recovery beyond what can be achieved by viscosity effects alone. As polymer injection continues, oil production gradually declines toward 2.3 PV, reflecting the progressive depletion of mobilizable oil.

During the post-flush waterflooding stage (1.8–2.3 PV), a slight additional increase in oil recovery is observed, reaching approximately 62%, corresponding to an incremental gain of about 1–2% of OOIP. Water cut stabilizes again at high levels (~90–100%). This additional recovery indicates that the polymer slug has durably modified the flow field, allowing subsequent water injection to mobilize residual oil in zones that were previously poorly swept.

Accordingly, the results demonstrate that high molecular weight polymer flooding significantly enhances oil recovery in heterogeneous reservoirs. The total incremental recovery is primarily achieved during the polymer injection stage (~32% OOIP), with a smaller contribution during post-flush waterflooding (~1–2% OOIP). This improvement is governed by a combined mechanism involving (i) enhanced macroscopic sweep efficiency due to mobility control and flow diversion, and (ii) improved microscopic displacement efficiency associated with viscoelastic effects. These findings are consistent with established theoretical and experimental studies [23,47,56].

#### 2.6.3. Effect of Low-Molecular-Weight Polymer Injection on Heterogeneous Oil Reservoirs

**Figure 9:**
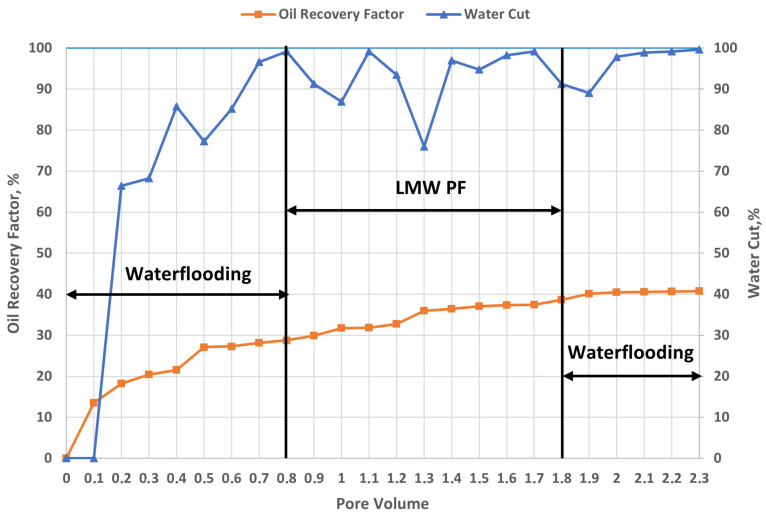
Oil recovery factor and water cut response during Flopaam 3230S flooding (T = 57 °C).

Figure 9 presents the evolution of the oil recovery factor and water cut as a function of injected pore volumes (PV), allowing the assessment of displacement efficiency when using a low molecular weight polymer (HPAM 3230S) in a heterogeneous reservoir. It should be emphasized that the initial waterflooding stage (0–0.8 PV) was conducted under conditions strictly identical to those used in the previously presented experiments.

Upon transition to polymer injection (0.8–1.8 PV) using HPAM 3230S, only a moderate improvement in displacement performance is observed. The oil recovery factor increases from ~29% to ~38–39%, corresponding to an incremental recovery of approximately 9–10% of OOIP during polymer flooding. Meanwhile, water cut remains high (≈85–100%) and exhibits notable fluctuations, indicating the persistence of preferential flow through high-permeability zones. However, a distinct behavior is observed in the low-permeability core: oil production resumes from approximately 1.0 PV and continues up to 2.3 PV, indicating partial flow diversion toward less permeable regions. Notably, at around 1.3 PV, oil production from the low-permeability core exceeds that of the high-permeability core, which corresponds to a temporary drop in water cut (from approximately ~95% to ~75–80%). This peak can be interpreted as a transient improvement in sweep efficiency due to partial flow redistribution.

This behavior can be explained by the physicochemical characteristics of the injected polymer. The relatively small molecular size of the low molecular weight polymer allows it to propagate more easily through both high- and low-permeability pore networks without creating significant flow resistance in the larger pore channels. Consequently, the contrast in flow resistance between high- and low-permeability zones remains insufficient to ensure effective mobility control. In addition, the polymer coil size remains small relative to the pore throat diameters of both cores, limiting its ability to selectively reduce permeability in high-permeability channels. As a result, only partial and temporary diversion of flow occurs. Furthermore, unlike high molecular weight polymers, this system does not exhibit significant viscoelastic effects, and therefore lacks additional microscopic displacement mechanisms such as elastic pulling or deformation of trapped oil ganglia.

During the post-waterflooding stage (1.8–2.3 PV), only a slight additional increase in oil recovery is observed, reaching approximately 41%, corresponding to an incremental gain of about 1–2% of OOIP. Water cut remains high (~90–100%), indicating that the flow profile has not been durably modified. Although oil production from the low-permeability core continues during this stage, the effect remains limited, and the system progressively returns to a regime dominated by high-permeability flow paths.

Thus, the results demonstrate that the use of low molecular weight polymer leads to only a modest improvement in oil recovery, primarily due to limited mobility control. The total incremental recovery remains relatively low compared to high molecular weight polymers, as both sweep efficiency and microscopic displacement efficiency are only marginally enhanced. The absence of significant viscoelastic properties, combined with the relatively small polymer coil size compared to pore throat dimensions, restricts its ability to effectively block high-permeability channels and mobilize residual oil. This limitation is particularly critical in heterogeneous reservoirs, where efficient conformance control is required to achieve substantial recovery improvements [47].

#### 2.6.4. Effect of Combined High- and Low-Molecular-Weight Polymer Injection on Heterogeneous Oil Reservoirs

**Figure 10:**
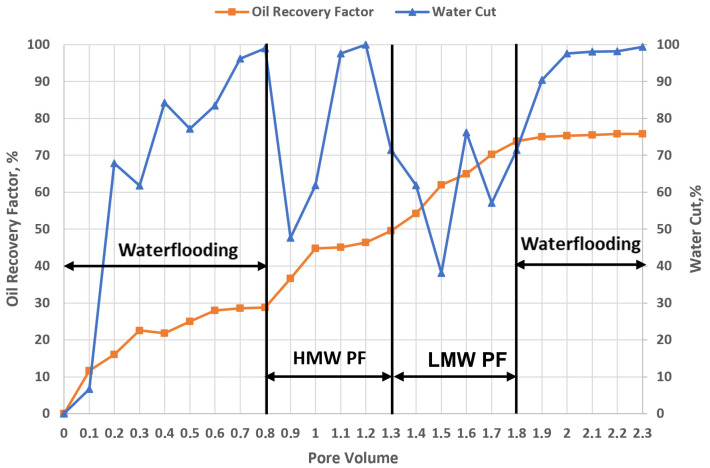
Oil recovery factor and water cut response during combined HPAM flooding (Flopaam 3630S + Flopaam 3230S) (T = 57 °C).

Figure 10 illustrates the evolution of the oil recovery factor and water cut as a function of injected pore volumes (PV) for a polymer flooding strategy combining high molecular weight (HMW) HPAM 3630S followed by low molecular weight (LMW) polymer injection. The initial stage (0–0.8 PV) corresponds to baseline waterflooding performed under identical conditions to previous experiments. During this period, oil recovery increases to approximately 28–29%, while water cut rapidly rises to ~90–100%, reflecting early water breakthrough and dominant flow through high-permeability channels, characteristic of an unfavorable mobility ratio and strong reservoir heterogeneity.

Upon initiation of HMW polymer injection (0.8–1.3 PV, 0.5 PV slug), a noticeable improvement in displacement efficiency is observed. Oil recovery increases from ~29% to ~50%, corresponding to an incremental recovery of approximately 21% OOIP during this stage. Simultaneously, water cut shows temporary reductions and fluctuations, indicating partial mobility control and diversion of the injected fluid toward previously unswept zones. The relatively large molecular size and viscoelastic properties of HPAM 3630S enhance flow resistance in high-permeability channels, promoting improved sweep efficiency and partial blocking effects.

A transition occurs at 1.3 PV with the injection of low molecular weight polymer (1.3–1.8 PV, 0.5 PV slug). During this stage, oil recovery continues to increase, reaching approximately 74%, corresponding to an additional incremental recovery of ~24% OOIP. The LMW polymer, characterized by smaller molecular size and absence of significant viscoelastic effects, is able to propagate more deeply into the pore network, including lower-permeability regions. This leads to a secondary redistribution of flow and mobilization of oil that was not accessed during the HMW polymer stage. However, despite its limited capacity to control mobility and block highly permeable channels, the overall improvement is remarkable. Water cut drops to approximately 38% at 1.5 PV with a slight fluctuation before rising again to 76%, reflecting the persistence of preferential flow paths.

During the post-flush waterflooding stage (1.8–2.3 PV), oil recovery increases slightly to approximately 76%, corresponding to an additional gain of ~2% OOIP. Water cut stabilizes again at high values (~90–100%), indicating that the flow field is still largely governed by high-permeability pathways.

In essence, the combined polymer injection strategy results in a total oil recovery of approximately 76%, representing a cumulative incremental recovery of ~44–45% OOIP beyond baseline waterflooding. The results highlight that the initial HMW polymer slug contributes primarily to mobility control and sweep improvement, while the subsequent LMW polymer injection provides limited additional recovery through deeper penetration into less accessible pore regions. However, the absence of sustained viscoelastic effects during the second stage, combined with the relatively short polymer slugs, limits the overall efficiency of the process. These findings suggest that while sequential polymer injection can improve recovery, optimal design of molecular weight, slug size, and injection sequence is critical to maximize both macroscopic sweep and microscopic displacement efficiency in heterogeneous reservoirs.

## 3. Conclusions

A series of core flooding experiments was conducted to evaluate the in-situ behavior of partially hydrolyzed polyacrylamide solutions in porous media. The effects of polymer molecular weight, concentration, and mechanical degradation on in-situ rheological properties were systematically investigated to optimize polymer injectivity and displacement performance. Based on the results, the following conclusions could be made:

1. Rheological behavior:

HPAM solutions exhibited strong shear-thinning behavior over the range 1–100 s^−1^, with viscosity increasing significantly with polymer concentration and molecular weight. The high-molecular-weight polymer (3630S) showed superior viscosity, particularly at low shear rates relevant to reservoir conditions.

2. Viscoelastic properties:

Only the high-molecular-weight polymer exhibited measurable viscoelasticity, characterized by G′ > G″ within the linear viscoelastic region and a well-defined relaxation time. The low-molecular-weight polymer showed predominantly viscous behavior (G″ > G′) with no elastic network formation.

3. Effect of physicochemical parameters:

Relaxation time increased with pore size and exhibited a non-monotonic dependence on salinity, with a maximum at intermediate NaCl concentration (~1–2 wt%). Increasing temperature reduced relaxation time due to enhanced molecular mobility.

4. Waterflooding performance (baseline):

Conventional waterflooding recovered approximately 28–29% OOIP, with early water breakthrough occurring at ~0.25–0.35 PV, leading to rapid water cut increase (60–80%) and poor sweep efficiency due to heterogeneity and unfavorable mobility ratio.

5. High-molecular-weight polymer flooding:

Injection of HPAM 3630S significantly improved recovery to ~61–62% OOIP, corresponding to an incremental recovery of ~32% OOIP. This improvement is attributed to enhanced mobility control, flow diversion, and viscoelastic effects promoting oil mobilization.

6. Low-molecular-weight polymer flooding:

Injection of HPAM 3230S resulted in limited improvement, with total recovery reaching ~38–41% OOIP (~9–10% OOIP incremental). The absence of viscoelasticity and reduced molecular size limited mobility control and conformance efficiency.

7. Combined polymer injection strategy:

Sequential injection (0.5 PV high MW + 0.5 PV low MW) achieved the highest recovery of ~74–76% OOIP, corresponding to an incremental gain of ~44–45% OOIP beyond waterflooding. The improvement results from the combined effects of mobility control (high MW) and deeper penetration (low MW).

8. EOR implications:

The results demonstrate that polymer molecular weight and viscoelasticity are key parameters controlling both macroscopic sweep efficiency and microscopic displacement efficiency in heterogeneous reservoirs. Proper optimization of polymer properties and injection strategy is essential to maximize oil recovery.

## 4. Materials and Methods

### 4.1. Polymer Solution Preparation

**Figure 11:**
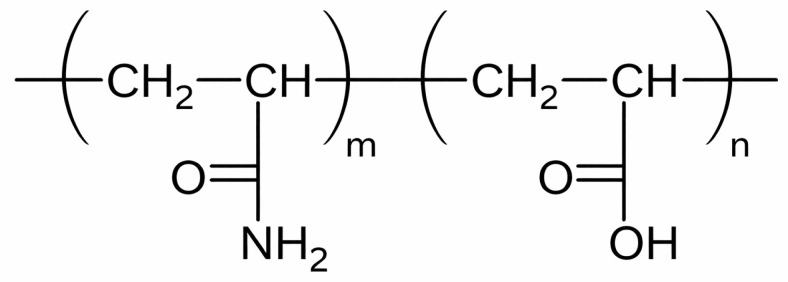
Chemical structure of partially hydrolyzed polyacrylamide (HPAM).

Two partially hydrolyzed polyacrylamide (HPAM) polymers (as illustrated in Figure 11), Flopaam 3630S and Flopaam 3230S (SNF Floerger, Andrézieux-Bouthéon, France), were investigated. Flopaam 3630S is an anionic polyelectrolyte characterized by a high weight-average molecular weight (Mw) in the range of 18–20 × 10^6^ Da and a moderate degree of hydrolysis (~30%), rendering it suitable for high-salinity applications with moderate hardness [57,58]. In contrast, HPAM 3230S exhibits a lower Mw in the range of 6–8 × 10^6^ Da, with a similar degree of hydrolysis (~30%). Both polymers were prepared following an identical protocol to ensure consistency.

For rheological measurements, polymer solutions were prepared in 3 wt% NaCl brine (30,000 ppm) using distilled water to simulate saline conditions. For core flooding experiments, formation water matching the target reservoir composition (Table 1) was used to better represent in-situ conditions.

A 10,000 ppm stock solution was prepared by gradually adding polymer powder to the vortex of stirred brine at 25 ± 1 °C, followed by mixing at 80 rpm for 4 h to ensure complete hydration while minimizing mechanical degradation. The solution was then aged for at least 12 h (overnight). Working solutions (5000 and 7500 ppm) were obtained by dilution with the same brine under gentle stirring, and the stock solution was also used for comparative rheological analysis.

### 4.2. Oil

Preliminary characterization of crude oil properties is required prior to laboratory and core flood experiments. The oil used in this study was obtained from the target reservoir (oil field under investigation) and is classified as a heavy crude, with a viscosity of 477 mPa·s at 25 °C and a density of 0.917 g/cm^3^.

In polymer flooding (EOR), the viscosity ratio between the injected polymer solution and crude oil governs the mobility ratio and displacement efficiency. To replicate reservoir conditions, the oil was heated to 57 °C, reducing its viscosity to approximately 75 mPa·s. All displacement experiments were conducted at this temperature.

### 4.3. Polymer Shear Characterization

The viscometric properties of the prepared partially hydrolyzed polyacrylamide solutions were quantitatively assessed using rotational rheometry to determine their flow behavior under simulated reservoir flow conditions.

**Figure 12:**
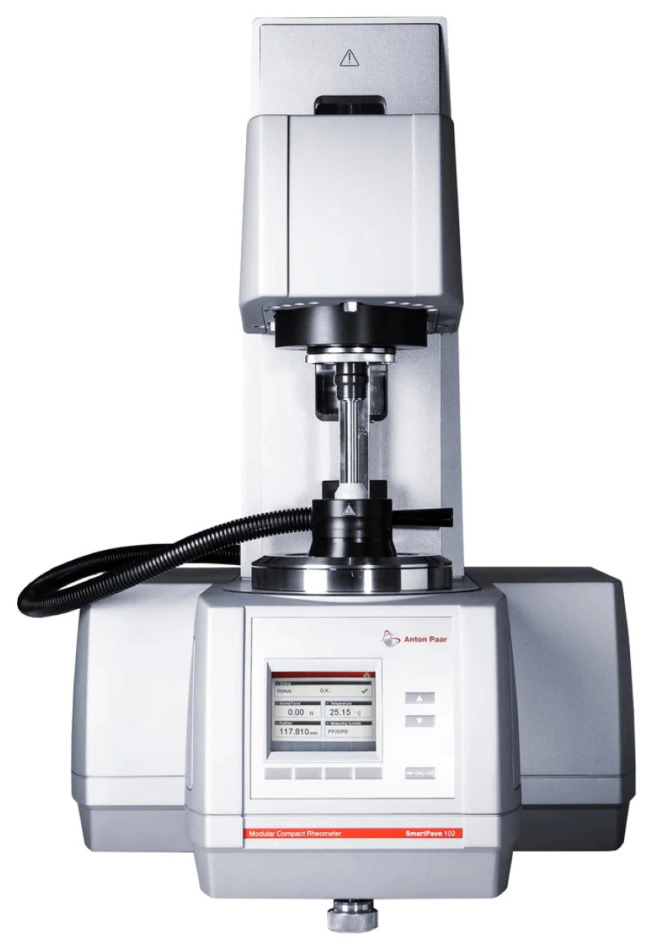
Anton Paar MCR 102 rotational rheometer.

A controlled-stress rheometer (MCR 102, Anton Paar GmbH, Graz, Austria) was employed for all measurements (Figure 12) [59,60,61]. The instrument was configured with a plate-plate measuring geometry, selected for its homogeneous shear field and suitability for low to medium viscosity fluids. The upper rotating plate and the lower stationary Peltier plate maintained a consistent gap to minimize edge effects and ensure accurate shear rate definition.

All experiments were conducted under isothermal conditions at 25.0 ± 0.1 °C, maintained by an integrated Peltier temperature control system, ensuring thermal equilibrium and data reproducibility consistent with the solution preparation temperature.

The steady-shear flow behavior was characterized through a controlled shear rate (CSR) sweep. The apparent viscosity (η) was measured across a shear rate ($\dot{\gamma }$) range of 1 to 100 s^−1^, encompassing the typical spectrum from near-quiescent conditionsŷ to high-shear scenarios encountered in near-wellbore flow and porous media.

### 4.4. Polymer Viscoelastic Characterization

The viscoelastic properties of polymer solutions are quantitatively described by the elastic (storage) modulus (G′) and the viscous (loss) modulus (G″), which represent the solid-like and liquid-like components of the material’s response, respectively.

A comprehensive oscillatory shear protocol was executed using the MCR 102 rheometer with a plate-plate geometry, maintaining an isothermal condition of 25.0 ± 0.1 °C. The protocol consisted of two sequential tests designed to probe the linear viscoelastic response and the frequency-dependent relaxation spectrum.

An amplitude (stress) sweep test was first conducted to identify the regime in which the microstructure of the polymer solution remains undeformed. A constant angular frequency (ω = 10 rad/s, selected as a representative value) was applied while the oscillatory shear stress (τ) was logarithmically incremented. The evolution of G′ and G″ was monitored as a function of applied stress. The limit of the Linear Viscoelastic Range (LVER) was defined as the critical stress (τ_c_) at which a deviation of more than 5% from the constant plateau value of G′ was observed. The corresponding critical strain (γ_c_) provided the maximum deformation applicable without inducing non-linear structural breakdown. In addition, the amplitude sweep test can be used to estimate the mechanical strength of the gel network. Within the linear viscoelastic region, the selected strain amplitude (γ = 10%) provides relevant information on the resistance of the gel structure and its ability to withstand deformation without structural damage. For the characterized polymers solutions, a strain amplitude of γ = 10% was determined to lie safely within the LVER and was subsequently adopted for frequency-dependent analysis.

Utilizing the strain amplitude (γ = 10%) established from the LVER, a frequency sweep was performed over an angular frequency range of 1 to 1000 rad/s. This test measures G′(ω) and G″(ω), mapping the material’s response from long-time (low-frequency) relaxation processes to short-time (high-frequency) dynamics.

The angular frequency at which G′(ω_c_) = G″(ω_c_) is call Crossover Frequency (ω_c_). This point signifies a transition from viscous-dominated (G″ > G′) to elastic-dominated (G′ > G″) behavior as timescale decreases.

A principal relaxation time, inversely proportional to the crossover frequency, was calculated as:

(1) $$\lambda =\frac{1}{\omega_{c\;}},$$

This parameter characterizes the longest relaxation mode of the entangled polymer network and is a critical indicator of the solution’s elastic memory.

### 4.5. Experimental Procedure

A typical 1D core flooding experiment consisted of several stages, including system validation, air permeability testing, and pressure control. Prior to fluid injection, all components were checked to ensure system integrity and repeatability of the measurements.

#### 4.5.1. Core Flooding Setup

A series of one-dimensional (1D) core flooding experiments were performed using a laboratory-scale system designed to simulate permeability contrasts typical of heterogeneous terrigenous reservoirs. The setup consisted of two parallel core holders representing high- and low-permeability zones. The main characteristics of the core samples are summarized in Table 2.

**Figure 13:**
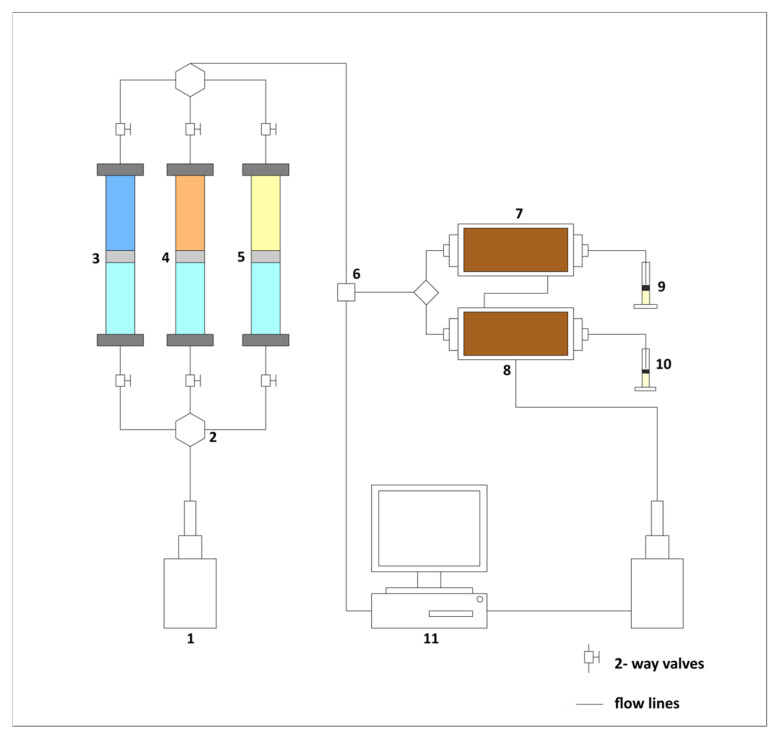
Schematic diagram of core flooding tester: (1) syringe pump, (2) six-way valve, (3) synthetic brine, (4) high molecular weight, (5) low molecular weight, (6) pressure sensor, (7) core holder (HMW), (8) core holder (LMW), (9) outlet (HMW), (10) outlet (LMW), (11) data acquisition system.

The system was connected to high-precision injection pumps and pressure sensors to monitor injection pressure and differential pressure across the cores. Produced fluids were collected using graduated receivers for accurate measurement. The maximum operating pressure of the system was 5000 psi, although all experiments were conducted well below this limit to ensure safe operation. A schematic of the experimental setup is provided in Figure 13.

#### 4.5.2. Waterflooding

Waterflooding was conducted as a secondary oil recovery method. After establishing an initial oil saturation of S_oi_ = 0.8, 0.8 pore volumes (PV) of brine were injected into the core samples. This process resulted in the recovery of approximately 30% of the original oil in place, leaving a significant amount of residual oil, which justified the implementation of polymer flooding as a tertiary recovery method.

#### 4.5.3. Polymer Flooding

Polymer flooding was performed following waterflooding to enhance oil recovery. Three injection scenarios were investigated: (i) injection of a high molecular weight polymer (1 PV), (ii) injection of a low molecular weight polymer (1 PV), and (iii) a combined strategy consisting of 0.5 PV of high molecular weight polymer followed by 0.5 PV of low molecular weight polymer. All polymer solutions were prepared at a concentration of 5000 ppm, which was selected based on its higher relaxation time compared to other concentrations investigated, indicating enhanced viscoelastic behavior. Polymer solutions were injected under reservoir representative conditions, with the temperature maintained at 57 °C. The injection rate was fixed at 1 cm^3^/min (1 cc/min) to ensure stable flow conditions, improve sweep efficiency, and minimize viscous fingering.

Effluent samples were collected continuously at the outlet, with collection vials replaced every 2.1 mL. The injection process was continued until the target polymer volume was fully introduced into the pore space.

#### 4.5.4. Post-Polymer Waterflooding

To maximize oil recovery and reduce residual oil saturation, an additional waterflooding stage was performed after polymer injection. Based on both literature and experimental observations, an injection volume of 0.5 PV was applied, which was found to be sufficient to achieve optimal displacement efficiency while limiting excessive water production.

## Figures and Tables

**Table 1:** Compositions of formation water.

Salt	Weight (g) per 1 L of Water
CaCl_2_·6H_2_O	4.5915
MgCl_2_·6H_2_O	0.80287
NaCl	17.6308
Na_2_SO_4_	0.00859
NaHCO_3_	0.41991

**Table 2:** Parameters of the cores used.

Core Name	Core Type	Length (cm)	Diameter (cm)	Porosity (%)	Gas Permeability (mD)
1777394228	sandstone	6.97	2.99	22.14	63.78
177769332	sandy siltstone	6.61	2.99	25.68	160.77

## Data Availability

Data are available on request due to restrictions, e.g., privacy or ethical. The data presented in this study are available on request from the corresponding author.

## Nomenclature

EOR	Enhanced Oil Recovery
G′	elastic (storage) modulus
G″	viscous (loss) modulus
HPAM	partially hydrolyzed polyacrylamide
HMW	high molecular weight
LMW	low molecular weight
Mw	molecular weight (MDa)
PF	Polymer Flooding
PV	pore volume, dimensionless
Q	injection flow rate (cc/min)
$\dot{\gamma }$	shear rate (s^−1^)
